# Cephalopods as Vectors of Harmful Algal Bloom Toxins in Marine Food Webs

**DOI:** 10.3390/md11093381

**Published:** 2013-09-06

**Authors:** Vanessa M. Lopes, Ana Rita Lopes, Pedro Costa, Rui Rosa

**Affiliations:** 1 Guia Marine Laboratory, Center of Oceanography, Faculty of Sciences, University of Lisbon, Av. Nossa Senhora do Cabo, 939, Cascais 2750-374, Portugal; E-Mails: vanessamadeiralopes@gmail.com (V.M.L.); anarjlopes@hotmail.com (A.R.L.); 2 IPMA—Portuguese Institute for the Sea and Atmosphere, Avenida de Brasília, Lisboa 1449-006, Portugal; E-Mail: prcosta@ipma.pt

**Keywords:** marine toxins, harmful algal bloom, cephalopods, *Octopus vulgaris*, *Dosidicus gigas*, *Sepia officinalis*, strandings

## Abstract

Here we summarize the current knowledge on the transfer and accumulation of harmful algal bloom (HAB)**-**related toxins in cephalopods (octopods, cuttlefishes and squids). These mollusks have been reported to accumulate several HAB-toxins, namely domoic acid (DA, and its isomers), saxitoxin (and its derivatives) and palytoxin (and palytoxin-like compounds) and, therefore, act as HAB-toxin vectors in marine food webs. Coastal octopods and cuttlefishes store considerably high levels of DA (amnesic shellfish toxin) in several tissues, but mainly in the digestive gland (DG)—the primary site of digestive absorption and intracellular digestion. Studies on the sub-cellular partitioning of DA in the soluble and insoluble fractions showed that nearly all DA (92.6%) is found in the cytosol. This favors the trophic transfer of the toxins since cytosolic substances can be absorbed by predators with greater efficiency. The available information on the accumulation and tissue distribution of DA in squids (e.g., in stranded Humboldt squids, *Dosidicus gigas*) is scarcer than in other cephalopod groups. Regarding paralytic shellfish toxins (PSTs), these organisms accumulate them at the greatest extent in DG >> kidneys > stomach > branchial hearts > posterior salivary glands > gills. Palytoxins are among the most toxic molecules identified and stranded octopods revealed high contamination levels, with ovatoxin (a palytoxin analogue) reaching 971 μg kg^−1^ and palytoxin reaching 115 μg kg^−1^ (the regulatory limit for PlTXs is 30 μg kg^−1^ in shellfish). Although the impacts of HAB-toxins in cephalopod physiology are not as well understood as in fish species, similar effects are expected since they possess a complex nervous system and highly developed brain comparable to that of the vertebrates. Compared to bivalves, cephalopods represent a lower risk of shellfish poisoning in humans, since they are usually consumed eviscerated, with exception of traditional dishes from the Mediterranean area.

## 1. Introduction

Primary producers constitute the basis of marine food-webs and phytoplanktonic blooms are typically beneficial to food-web processes. However, under certain conditions, harmful algal blooms (HABs), a natural phenomenon, can become harmful to other forms of life. HABs become harmful through two types of mechanisms: non-chemical and chemical. The former have negative effects on marine life due to the sheer number of microalgae cells occupying the ocean, creating an anoxic barrier between the surface and deeper waters. The latter act through the production of marine phycotoxins, and other metabolites [1]. Out of the 5000 species of phytoplankton known today, about only 2% of them are toxin producers [2,3]. Toxins may cause heavy damage to marine animals and even death (e.g., behavior alterations [4], development impairments in early stages of life [5,6], abortion and premature birth of marine mammals [7]). They can also affect human populations, through the consumption of contaminated sea products, mostly shellfish [2]. As a result, many coastal countries conduct monitoring programs for marine toxins in shellfish [2].

**Figure 1:**
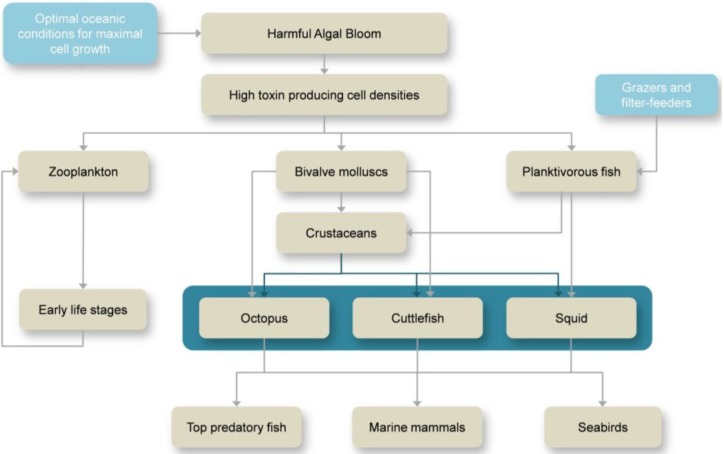
Routes of exposure and transfer of harmful algal bloom (HAB) toxins through marine food webs.

The exposure pathways of aquatic animals to marine toxins (see example in Figure 1) depend on the: (i) Ecology of the toxin producer (e.g., pelagic, epibenthic); (ii) Environmental conditions at the time of the bloom; and (iii) Probability of the organism to become in contact with the toxin. Marine organisms can come in direct contact with toxin-producing microalgae by ingestion of the cells (e.g., filter feeders). Yet, some microalgae species produce toxic exudates that are released into the water and come in contact with other organisms [8,9]. The persistence of the toxin in the water depends on the physical and chemical properties of seawater, as well as the volume of water where the toxin has been released, *i.e*., larger volumes of water tend to dilute the toxin and reduce significantly the risk of contamination. The same applies for lysed toxin-producing microalgae. When the bloom becomes senescent or when seawater properties are no longer optimal for cell growth, microalgae die and as their cells lyse the toxins within are released to the surrounding environment.

Marine toxins are transferred to higher levels of the food web by predation on organisms that have been in direct contact with the toxins (Figure 1) and have accumulated, converted or amplified the effect of the toxin, thus, creating a chain of vectors of the toxin. Some organisms convert the toxin into less toxic compounds, others magnify its toxicity [2]. This way, the toxin produced by microscopic algae can be passed onto the top predators of the marine food web (and even humans) and cause events of mass mortality [10,11,12,13,14,15,16,17,18]. The amount of toxin accumulated in the organism’s system is dependent on: (i) The solubility (hydrophilic or lypophilic) of a given toxin; (ii) The capability of the organism to excrete/detoxify it effectively; and (iii) The organism’s physiological tolerance (see [2] and references therein).

Cephalopods occupy an important position in the marine food web. They are known to be preyed upon by marine mammals [19,20,21], top predatory fish [22,23] and seabirds [24]. On the other hand, they prey mostly on crustaceans, small fish and other mollusks, which are known vectors of marine phycotoxins [25,26,27]. Although these organisms play a key role in the link between primary production and higher trophic levels, there are still few studies on the transference and accumulation of marine toxins. This review summarizes, for the first time, the current knowledge of HAB-related toxins in these highly versatile and opportunistic mollusk predators.

## 2. Overview on HABs and Marine Toxins

**Figure 2:**
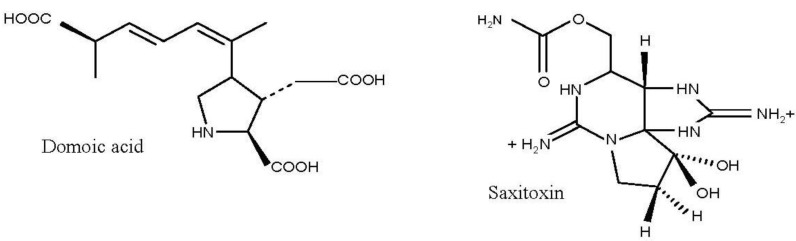
Structures of domoic acid and saxitoxin.

One of the most studied marine toxins is domoic acid (DA, Figure 2, Table 1), a water soluble non protein amino acid responsible for amnesic shellfish poisoning (ASP). It acts as an analogue of glutamic acid and binds irreversibly to glutamate receptor sites, causing destructive neuronal depolarization [28] and permanent short-term memory loss in mammals [29,30]. This neurotoxin is produced by a group of diatom species comprising both *Pseudo-nitzschia* and *Nitzschia* genera [31,32,33,34,35]. DA has been pointed out to be one of the main toxins responsible for events of mass mortality of marine mammals [36,37] and seabirds [38,39] throughout the world, drastically endangering the delicate balance of the marine ecosystem. 

**Table 1:** Maximum levels of HAB-associated toxins in cephalopods.

Toxin	Species	Maximum levels found	Tissue/Organ	Reference
DA	*Octopus vulgaris*	166.2 μg DA g^−^^1^	Digestive gland	[40]
	*Eledone moschata*	127 μg DA g^−^^1^	Digestive gland	[41]
	*Eledone cirrhosa*	18.8 μg DA g^−^^1^	Digestive gland	[41]
	*Sepia officinalis*	241.7 μg DA g^−^^1^	Digestive gland	[42]
	*Doryteuthis opalescens*	0.37 μg DA g^−^^1^	Stomach	[43]
	*Dosidicus gigas*	0.23 μg DA g^−^^1^	Digestive gland	[44]
STXs	*Octopus vulgaris*	35 μg STX equivalents g^−^^1^	Digestive gland	[45]
	*Dosidicus gigas*	4.83 μg STX equivalents g^−^^1^	Digestive gland	[44]
	*Octopus* (*Abdopus*) sp. 5	2.46 μg STX equivalents g^−^^1^	Arms	[46]
PlTX	Octopod (species unknown)	971 μg/kg OVTX-a and 115 μg/kg PlTX	Unspecified	[47]

Mussels (*Mytilus edulis*) were the first organisms to be associated with ASP, after the death of 3 people and over 100 people becoming ill and suffering neurological damage in Prince Edward Island, Canada, in 1987 [30,48]. A regulatory limit of 20 μg DA g^−1^ shellfish meat was then established [49]. Since then, no other human mortalities have been reported, however, there have been other DA-poisoning events involving other organisms such as marine mammals and seabirds. In these cases, vectors of DA other than bivalves were identified, namely planktivorous crustaceans [50,51,52] and fish [53,54,55,56]. 

Other globally found marine toxins are the paralytic shellfish toxins (PST), which are produced by some dinoflagellate species belonging to three genera: *Gymnodinium*, *Pyrodinium* and *Alexandrium* [57], and are responsible for paralytic shellfish poisoning (PSP) events. There are a number of compounds belonging to the PST family and may be divided into three groups according to their chemical structure, namely: (i) carbamoyl (saxitoxin—STX, neosaxitoxin—NEO, gonyautoxins—GTX 1–4); (ii) decarbamoyl (derivatives of STX, NEO and GTX); and (iii) sulfamate (C-toxins 1–4, B1—GTX5, B2—GTX6) toxins. The most toxic group is the carbamoyl toxins [58], and among them, saxitoxin (STX, Figure 2, Table 1) is the most studied and toxic. It blocks the conduction of electrical impulses in axons, and consequently, impacts the nervous system and impairs sensorimotor function in vertebrates [5,17,59,60]. In fact, PSTs are responsible for the numerous deaths of humpback whales [13] and massive fish mortalities [15,16,17,18]. Regarding the latter vertebrate group, PSTs have been mostly detected in planktivorous species, like sardines (*Sardinops sagax*, *Sardina pilchardus*) [61,62], herring (*Clupea* sp.) [5,16,18], mackerel (*Scomber* sp.) [63], horse mackerel (*Trachurus trachurus*) [64] and anchovies (*Engraulis mordax*) [61]. PST episodes (*Alexandrium catenella* blooms) involving sea otters are not uncommon (in Monterey Bay, CA, USA) and studies suggest they have learnt to avoid preying on toxic shellfish [4]. PSTs have also been found in invertebrate groups, namely crustaceans [61]. 

The third main group of marine toxins causes diarrhetic shellfish poisoning (DSP). The toxins responsible are okadaic acid (OA) and its derivatives (dinophysistoxins—DTX 1–4) and are produced by the dinoflagellates *Prorocentrum lima* and a number of species belonging to *Dinophysis* genus. OA is a complex fatty acid that inhibits specifically the activity of protein phosphatases 1 and 2 without damaging other proteins [65] and thus it increases protein phosphorylation and causes gastrointestinal distress [2]. The knowledge of the interlinked ecology of DST-producing species and their consumers is scarce since success on the establishment, maintenance and growth cultures of *Dinophysis* has been limited [66]. 

Pectenotoxins (PTX) and yessotoxins (YTX) have similar targets and actions as DTXs and both have been first discovered in scallops. Pectenotoxins are produced by the dinoflagellate *Dinophysis* spp*.* [67] and YTXs are produced by *Protoceratium reticulatum*, *Lingulodinium polyedrum* [68,69] and *Gonyaulax spinifera* [70,71].

Azaspiracids (AZAs) are lipophilic polyether toxins that have been relatively recently discovered and once thought to be produced by the heterotrophic dinoflagellate *Protoperidinium crassipes*, but currently it is known to be produced by the dinoflagellate *Azadinium spinosum* [72]. This toxin causes similar effects to those of DST, but unlike DSTs, AZAs are known to cause neurological damaged as well as gastrointestinal damage in mice. The effects of this toxin in aquatic organisms remains poorly studied [2].

Spirolides (SPX) are macrocyclic spiroamines that have been recently discovered to be produced by the dinoflagellate *Alexandrium ostenfeldii*. There has been limited research on this marine toxin, but it is known that it inhibits the acetylcholine-induced calcium signal and due to persisting symptoms, is probably an irreversible antagonist of these receptors [73]. 

Neurotoxic shellfish poisoning (NSP) is an illness caused by the ingestion of contaminated shellfish which have fed on the dinoflagellate *Karenia brevis* (=*Gymnodinium breve*) and raphidophyte *Chatonella* sp., *Heterosigma akashiwo* and *Fibrocapsa japonica*, with the dinoflagellate being responsible for most cases of NSP [2]. These organisms produce brevetoxins (BTX) which are a group of complex polycyclic polyether compounds that bind to voltage sensitive sodium channels in nerve cells and cause alteration of the membranes in excitable cells [2,74] leading to interferences of normal processes in nerve cells [74]. This toxin is a known ichthyotoxin and is responsible for massive fish kills throughout the world [2]. Recently, one bloom event of *Chatonella* killed up to 2.5 billion Atlantic menhaden *Brevoortia tyrannus* in 3 months [10]. Fish kills are assumed to be caused by the absorption of the toxin through the gills when the bloom enters senescence and cells lyse releasing the toxin into the water [2]. Mortality of marine mammals, namely dolphins [14] and manatees [75] has also been reported, with the former being caused by trophic transfer from Atlantic menhaden and the latter was suspected to be affected through three possible routes: (i) toxic aerosol inhalation; (ii) toxic food ingestion; or (iii) toxic seawater ingestion. Additionally, in 1963 it was reported that an unusually large bloom of *K. brevis* caused sea turtles to strand in mass, with high mortality rates of cormorants and numerous species of fish [75]. 

The dinoflagellate, *Gambierdiscus toxicus*, is also responsible for the production of ciguatoxins (CTX), a toxin that causes ciguatera, an illness triggered by the ingestion of tropical fish contaminated with CTX. This toxin is a lipophilic sodium channel activator and like many of the others previously mentioned, binds to voltage sensitive sodium channels of nerve cells and excitable tissues [76].

**Figure 3:**
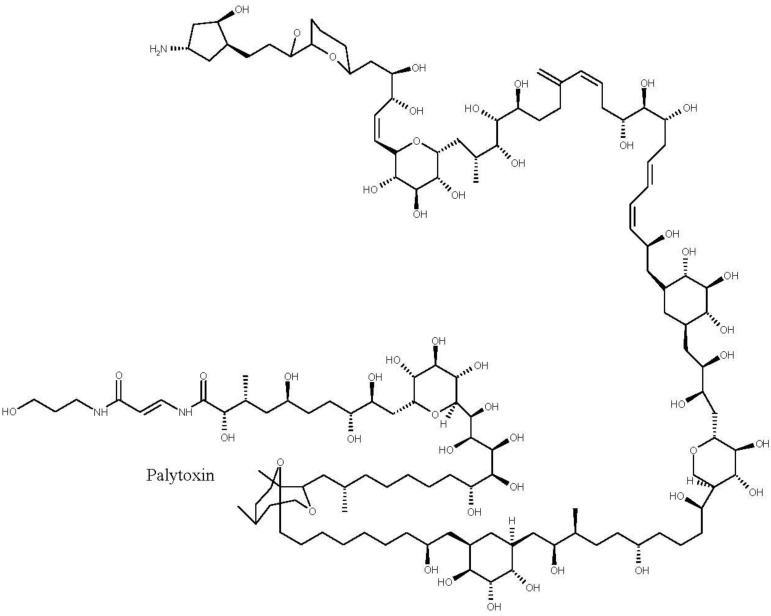
Structure of palytoxin.

Last, palytoxins (Figure 3) are considered one of the most lethal marine toxins [77] and are produced by benthic dinoflagellates of the genus *Ostreopsis.* They have the longest chain of continuous carbon atoms known to any natural substance and, thus, referred to as super-carbon-chain compounds [78]. There are reports of great animal mortalities following an outbreak of *Ostreopsis* spp., namely of sea urchins [79,80], cirripeds, bivalves, gastropods, other echinoderms and small fish [81,82]. Negative effects on benthic organisms such as limpets, mussels, balanids and sea urchins have been associated with high cell densities of *Ostreopsis*. One visible negative effect on sea urchins is the loss of their guard spines [79]. Although it is known the mode of action of palytoxin, which is a complex molecule that binds to sodium and potassium channels extracellularly inhibiting the active transport of sodium and potassium across the membranes leaving the channel permanently open and causing death by the excess of intercellular cations [83], little is known regarding adverse effects on benthic invertebrates [84]. 

## 3. Cephalopod Life Strategies and Feeding Ecology

**Figure 4:**
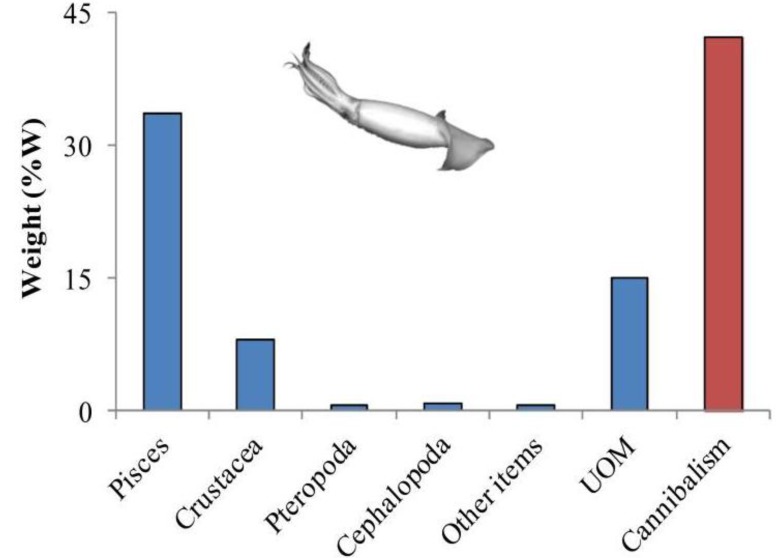
Weight (%W) of prey in the stomach contents of jumbo squid (*Dosidicus gigas*) from the Gulf of California during 1998–1999 (data from Markaida [94]). %W is defined as the weight of a certain prey relative to the total weight of all prey, expressed as a percentage. Legend: UOM—unidentified organic matter.

**Figure 5:**
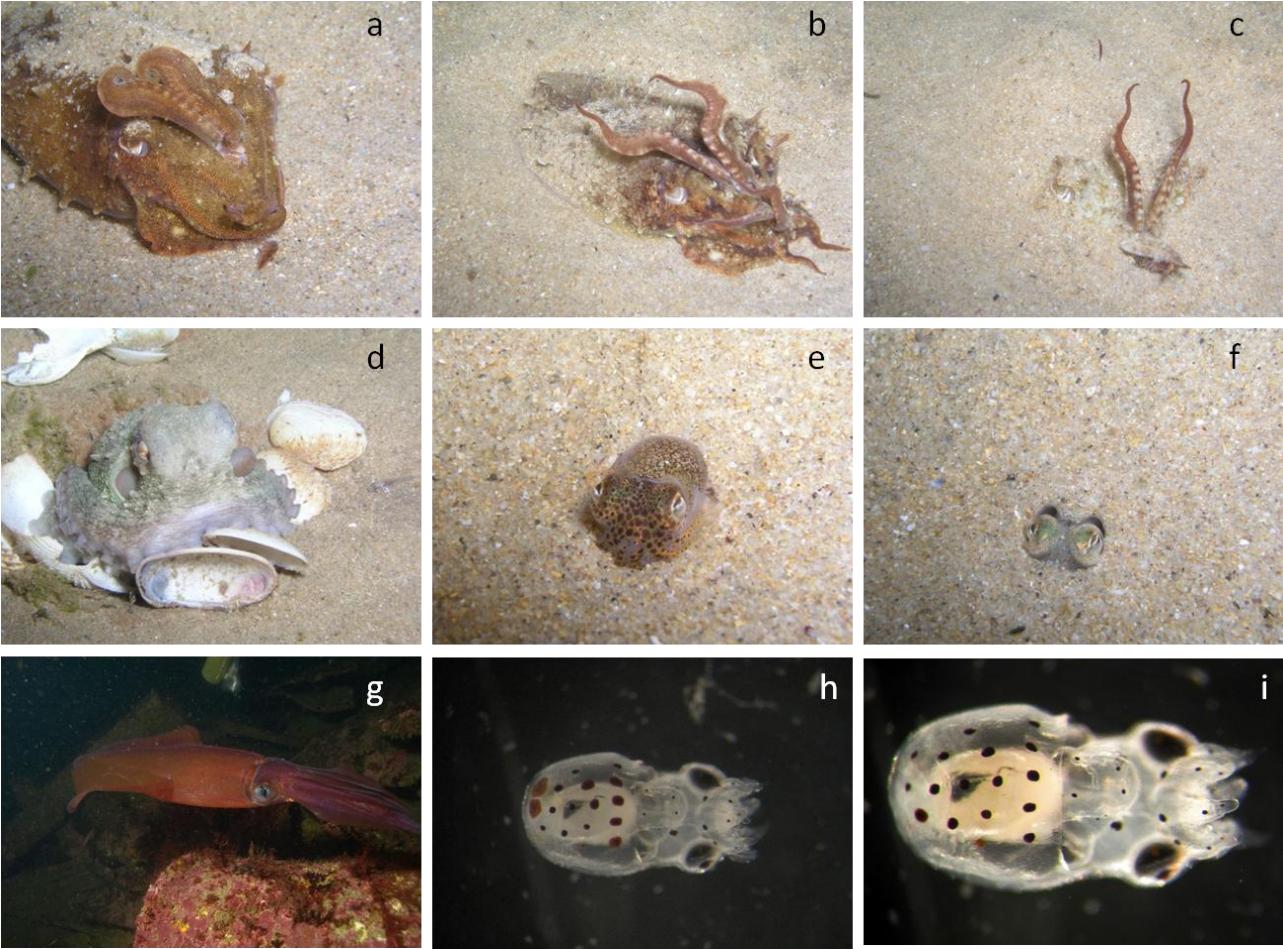
Cephalopod coastal diversity and respective life strategies. (**a**–**c**) nektobenthic common cuttlefish, *Sepia officinalis*; (**d**) benthic common octopus, *Octopus vulgaris*; (**e**,**f**) benthic sepiolid, *Sepiola atlantica*; (**g**) semi-pelagic squid, *Loligo vulgaris*; (**h**,**i**) planktonic paralarvae of common octopus, *Octopus vulgaris* (photo credits: Rui Rosa).

The class Cephalopoda is a very diverse group of exclusively marine mollusks that is currently represented by octopods, squids, sepiolids and cuttlefishes and the pre-historic *Nautilus*. There are around 800 species of cephalopods inhabiting the ocean from the poles to the tropics, from intertidal pools to the abyssal trenches, exhibiting various life strategies [85,86,87]. Cephalopods are considered to be the most evolved invertebrates, since they can be compared to vertebrates by many features, such as their panoply of complex behaviors, well-developed senses and highly developed nervous system with a complex brain [88,89,90,91]. They are also known for their ability to mimic the surrounding environment with changes in color and texture of their skin in order to avoid predators, to capture prey or even to communicate with each other [92]. Regarding the feeding ecology, cephalopods are voracious carnivores with many different feeding strategies (including cannibalism, Figure 4) that enable them to feed opportunistically on a wide range of prey (e.g., Table 2). Nektobenthic cephalopods such as cuttlefish, can swim in the water column through the constant undulation of the fins but spend considerable amounts of time buried in the sediment to rest or to ambush their prey (sit-and-wait strategy; Figure 5a–c). On the other hand, benthic cephalopod fauna, mostly represented by Octopoda, (Figure 5d) and *Sepiolida* species (Figure 5e,f), spend all of their lives on or near the bottom. 

**Table 2:** Diet of common octopus (*Octopus vulgaris*) in the Portuguese coast, namely in Viana do Castelo (North region), Cascais (Centre region) and Tavira (South region). Occurrence index (OCI) of prey found in the octopods’ stomach contents (data from Rosa *et al*. [93]).

Prey category	Occurrence index (OCI)		
	Viana	Cascais	Tavira
ANNELIDA	1.85	2.52	-
POLYCHAETA unidentified	1.85	2.52	-
CRUSTACEA	38.27	67.31	36.90
ISOPODA unidentified	-	-	0.18
DECAPODA NATANTIA	1.93	6.04	2.57
Natantia unidentified	1.93	3.43	2.57
Caridae unidentified	-	2.61	-
DECAPODA REPTANTIA	36.34	61.27	34.14
ANOMURA	-	-	0.33
Paguridae unidentified	-	-	0.26
*Pagurus prideauxi*	-	-	0.07
BRACHYURA	36.34	61.27	33.81
Brachiura unidentified	18.96	39.33	17.51
Portunidae unidentified	10.84	21.94	10.17
*Liocarcinus* sp.	5.17	-	5.03
*Polybius henzlow*	1.36	-	1.10
MOLLUSCA	7.13	14.89	23.52
GASTROPODA unidentified	1.49	1.87	0.61
BIVALVIA	1.75	7.43	16.56
Carditidae unidentified	-	-	1.07
Cardiidae unidentified	-	-	0.73
*Mytilus* sp.	-	-	1.72
*Venus* sp.	-	-	1.48
Bivalvia unidentified	1.75	7.43	11.56
CEPHALOPODA	3.90	5.59	6.35
Sepiolidae unidentified	-	-	1.39
*Sepia* sp.	-	-	0.73
*Illex coindetii*	0.71	-	-
*Octopus* sp.	0.18	0.83	0.36
Cephalopoda unidentified	3.01	4.75	3.87
OSTEICHTHYA	52.74	15.28	39.59
Clupeidae unidentified	16.70	3.29	12.94
Gobidae unidentified	5.80	4.53	1.43
Osteichthya unidentified	30.24	7.46	25.22

Coastal and shelf cephalopods feed primarily on fish, crustaceans and other cephalopods (schematic view presented in Figure 1). Although some studies point out that benthic species tend to prey mainly on crustaceans while fishes predominate the pelagic species [95,96,97], others studies indicate that the prey spectrum in the diet is related to the most readily available prey (Table 2) [93]. For instance, Smale and Buchan [98] and Ambrose and Nelson [99] showed that octopus had a greater preference for bivalves, forming up to 88% of its diet. In contrast, Altman [100], Nigmatullin and Ostapenko [101], Guerra [102], Sanchez *et al*. [103] and Quetglas *et al*. [104] found a large proportion of crab and other crustaceans, making up around 70% of the diet. It is worth noting that the data on the feeding habits of octopods are biased by the sampling method used, and the proportion of bivalves and gastropods found in the stomachs is usually underestimated. Contrarily, the studies based on debris found near the middens overestimate such food items (Figure 5d). 

After some external digestion, the flesh is consumed and the exoskeleton rejected. Newly hatched octopods (paralarval stage; Figure 5h,i) feed on planktonic crustaceans and when they settle to the seabed they switch the diet to nektobenthic crustaceans and benthic mollusks [105]. The juveniles of coastal squids (Figure 5g) and cuttlefish (Figure 5a–c) mostly prey on crustaceans but shift their diet to fish and cephalopods as they grow. The sepiolids (Figure 5e,f) feed almost exclusively on mysids and decapods crustaceans, neglecting crabs and fish [106]. 

In the oceanic region, in order to support their particular life-history (e.g., high growth rates, short lifespan, semelparity) and physiological traits (e.g., high metabolic rates associated with the energetically-inefficient jet propulsion), most pelagic squids are well adapted to the seasonality and spatial patchiness of food resources.

To maintain their growth and maturation rates, they are known to make extensive migrations to exploit the latitudinal differences in productivity [86,107,108]. Some oceanic squids also undertake sporadic (or recurrent) migrations over the continental shelves to feed (e.g., *Todarodes sagittatus*, *Dosidicus gigas*). Moreover, most of these squids exhibit daily vertical migrations, from the darker and colder waters of the mesopelagic zone to the warm, well lit and more productive epipelagic zone. One of the most well-known examples is the Humboldt squid, *Dosidicus gigas*, a large ommastrephid squid that lives around 300 m during the day and migrates to the surface at night to feed [109], mainly on planktivorous fish, like myctophids, anchovies and sardines [110]. It, thus, represents an important link in the exchange of energy throughout the water column. 

The wide variety of life (and feeding) strategies makes cephalopods an important link in the marine food web (Figure 1), by connecting the primary production to the higher trophic levels. Consequently, they also play a key role on the transference and accumulation of marine toxins. 

## 4. HAB-Toxins in Cephalopods

### 4.1. Amnesic Shellfish Toxins

**Figure 6:**
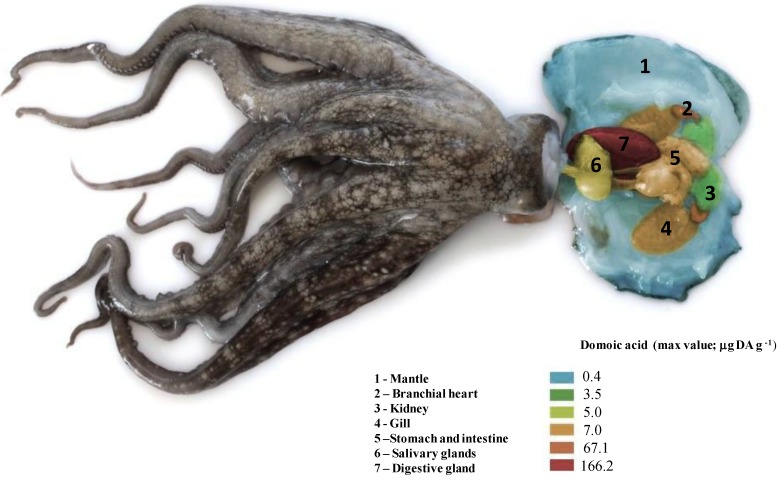
Schematic illustration of the maximum levels (μg g^−1^) of amnesic shellfish toxin, domoic acid (DA) found in common octopus (*Octopus vulgaris*) tissues.

**Figure 7:**
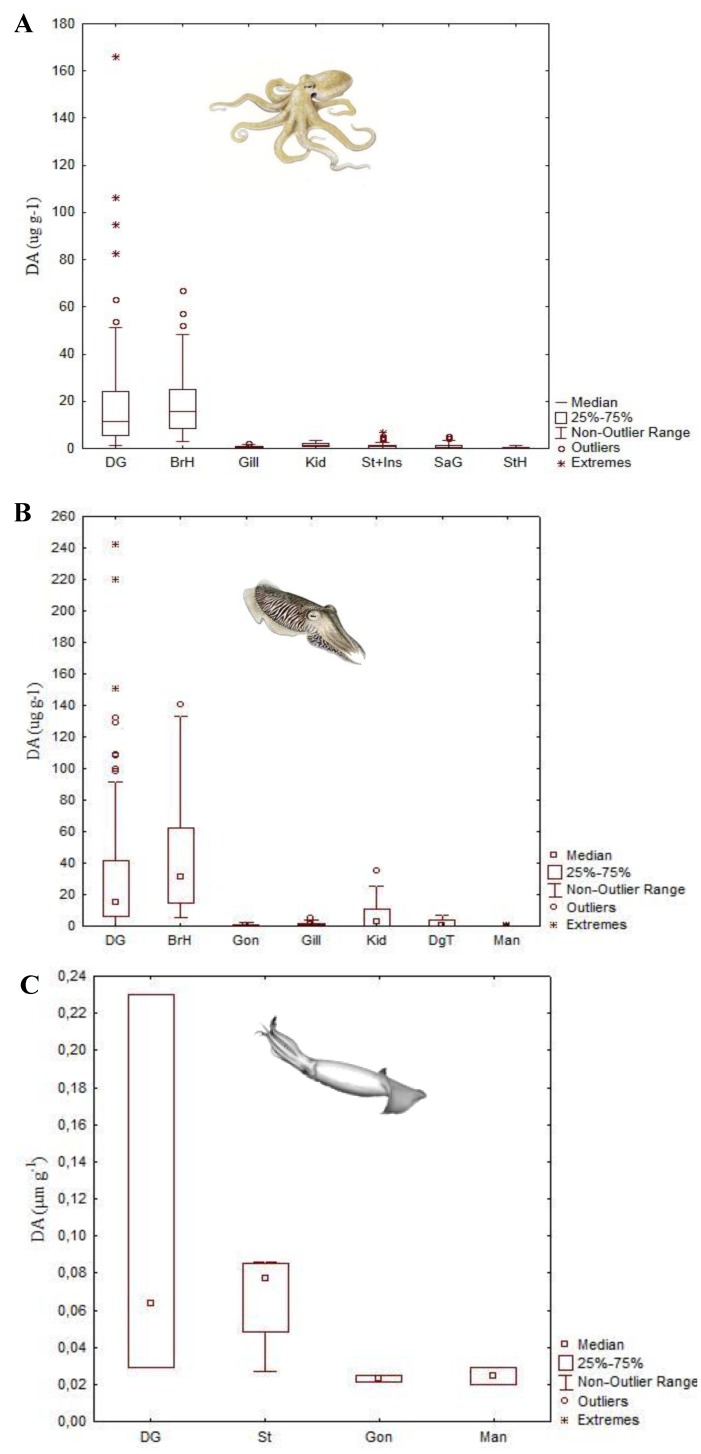
Domoic acid levels (DA, μg g^−1^; median, 25 and 75 quartiles, non-outlier range and outliers) detected in the tissues of: (**A**) Common octopus (*Octopus vulgaris*), collected in the NW and South Portuguese coast; (**B**) Common cuttlefish (*Sepia officinalis*), collected in the NW Portuguese coast; and (**C**) Humboldt squid (*Dosidicus gigas*) collected in British Columbia, Canada. Abbreviations: DG—digestive gland; BrH—branchial hearts; Gill—Gills; Kid—kidney; Gon—gonads; St+Inst—pooled stomach, caecum and intestine; SaG—posterior salivary glands; StH—systemic heart; DgT—digestive tract; Man—mantle (data from Costa *et al*. [40], Costa *et al*. [42] and Braid *et al*. [44]).

Non filter-feeding organisms accumulate DA through transfer from lower to higher trophic levels, since *Pseudo-nitzschia* diatoms can be preyed upon by both benthic and pelagic members of the food web [2]. Cephalopods have been reported to accumulate and store DA in their tissues (Figure 6, Figure 7). High variable DA levels, ranging from 1.1 to 166.2 μg DA g^−1^ were found in the digestive gland (DG) of the common octopus (*Octopus vulgaris*) off the Portuguese coast (Figure 7A; Table 1). The highest DA levels are observed in DG because this organ is the primary site of digestive absorption and intracellular digestion in cephalopods [93,111]. Thus, it may act as a DA reservoir, as it is for other substances (lipids, contaminants, *etc*.) [112]. Ontogenetic studies on the accumulation of DA in the DG of *O. vulgaris* also showed that younger octopus presented higher DA levels [113] due to their faster growth and food conversion rates [114]. It has also been shown that younger octopus choose smaller mussel size classes, which are known to concentrate higher levels of toxins [98]. Moreover, female octopus accumulate higher DA levels in the DG than males [113], probably because females have greater energy requirements (and subsequent higher metabolic and food conversion rates) for egg production [114]. Typically, DA levels are detected during, or shortly after, blooms of DA producing algae, but, although in relatively low concentrations (1.0 to 26.6 μg DA g^−1^). DA was detected in the DG of *O. vulgaris* 4 to 5 months after the bloom, suggesting a retention capability of DA in the octopus’ system for long periods of time [115].

**Figure 8:**
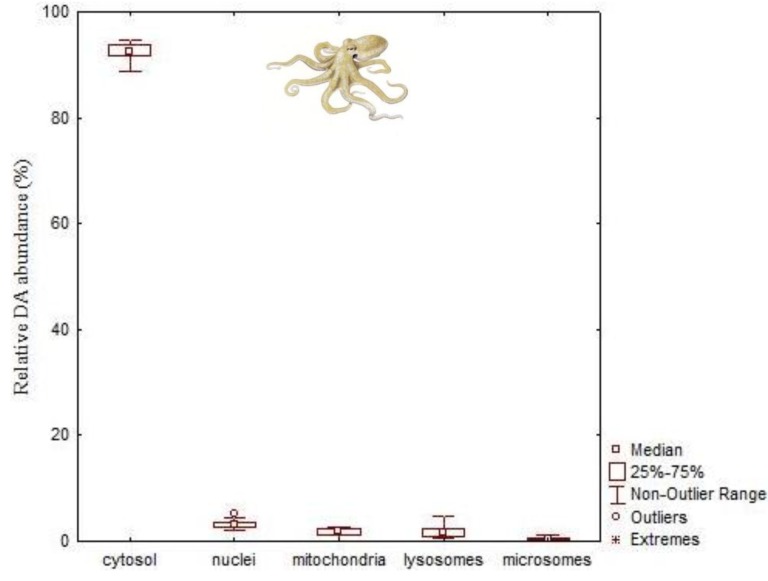
Domoic acid distribution in the DG cell fractions of the common octopus (*Octopus vulgaris*; median, 25 and 75 quartiles, non-outlier range and outliers) collected in the NW Portuguese coast (data from Lage *et al*. [115]).

Sub-cellular partitioning of DA in the soluble and insoluble fractions (nuclei, mitochondria, lysosome, microsome) showed that nearly all DA (92.6%) is found in cytosol (Figure 8), irrespective of toxin levels. The distribution of the remaining DA in each fraction was found in the following decreasing order: nuclei > lysosomes > mitochondria > microsomes [115]. This favors the trophic transfer of the toxins since cytosolic substances can be absorbed by predators with greater efficiency.

The branchial hearts also accumulate highly variable levels of DA, ranging from 3.0 to 67.1 μg DA g^−1^, surpassing, in some cases, the values detected for the DG (Figure 7A,B). The branchial hearts are located at the base of the gills, receiving deoxygenated blood from the body tissues. From them, blood is then sent to the gills where it is oxygenated. In addition to this pumping function, the branchial hearts have an excretory role [116,117]. Moreover, it has been demonstrated that these organs are able to accumulate high concentrations of some heavy metals [118,119,120], for this reason, they have also been called “kidneys of accumulation” [121]. However, DA concentrations in the kidney are substantially lower (0.2–3.5 μg DA g^−1^) (Figure 7A,B) [40]. 

**Figure 9:**
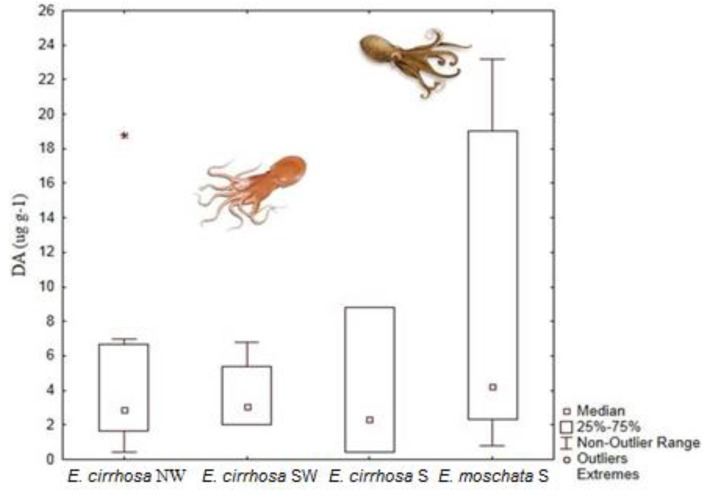
Domoic acid levels (DA; μg kg^−1^; median, 25 and 75 quartiles, non-outlier range and outliers) detected in the DG of *Eledone cirrhosa* (from NW, SW and South Portuguese coast) and *E. moschata* (from South Portuguese coast) (data from Costa *et al*. [41]).

Further studies were performed on horned octopus (*Eledone cirrhosa*), the musky octopus (*E. moschata*) and common cuttlefish (*Sepia officinalis*) off the Portuguese coast. Again, all these studies showed that the DG is the main organ of DA accumulation (Figure 7, Figure 9; Table 1) [40,41,42,113]. It is worth noting that DA levels in the DG of *E. moschata* were higher than in *E. cirrhosa* (ranging from 0.8 to 127 μg DA g^−1^; Figure 9). Although these species are very similar and their geographic and bathymetric range partially overlaps, *E. moschata* generally inhabits shallower waters in the Portuguese Southern coast and, therefore, has a different feeding regime [41]. The horned octopus feeds mainly in crustaceans, but other types of prey have been reported as well, like fish, cephalopods, polychaetes and gastropods [122,123,124]. Unfortunately, there is no available information about the diet of the musky octopus, except for laboratory studies that have shown that this octopus readily feeds on crabs, and thus the possible vector of DA remains unclear [125]. The only study performed on the common cuttlefish, *Sepia officinalis*, revealed high DA levels in the DG and branchial hearts, ranging from 1.1 to 241.7 μg DA g^−1^ and 5.1 to 140.8 μg DA g^−1^, respectively (Figure 7B). This study also showed that levels of DA persist through long periods of time after the bloom of *Pseudo-nitzschia*, and that the toxic profile suggests that there is degradation of DA into less neurotoxic isomers by the branchial hearts [42].

**Figure 10:**
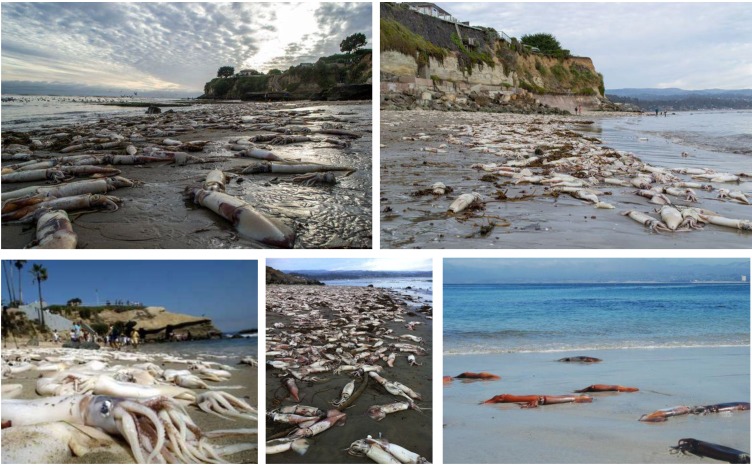
Stranded Humboldt squids (*Dosidicus gigas*) in Californian (top panels, and bottom left and middle panels) and Mexican coasts (bottom right panel).

The available information on the accumulation and tissue distribution of DA is very scarce for squid. The loliginid *Doryteuthis opalescens* has shown to accumulate much lower DA levels than the other cephalopod groups mentioned above. The highest levels of DA were found in the stomach (0.37 μg DA g^−1^), while the viscera (including DG) showed values around 0.10 and 0.19 μg DA g^−1^ [43]. Additionally, stranded Humboldt squids (Figure 10) have also been shown to present low levels of DA in the stomach, gonad, DG and mantle. As expected, the highest concentrations of DA were found in the DG (up to 0.23 μg DA g^−1^) (Figure 7C) [44]. For decades there have been records of periodic events of massive Humboldt squids (Figure 10), mostly juvenile specimens, stranding on the beaches of the Eastern Pacific Ocean. However, their frequency has increased and the geographical profile has changed, since there are now records from the Mexican shore to Alaska. One of the explanations for the occurrence of these strandings is the uptake of DA through consumption of planktivorous fish, like the pacific sardine, during blooms of *Pseudo-nitzschia* spp*.* [61]. This implies important cascading effects in the marine food web, since Humboldt squid make up a large portion of the diet of many top marine predators [126]. The Humboldt squid may act as DA vectors to dolphins, whales and sea lions populations, which may be in jeopardy considering the devastating DA effects in the mammalian nervous system [7,11,12,127].

### 4.2. Paralytic Shellfish Toxins

**Figure 11:**
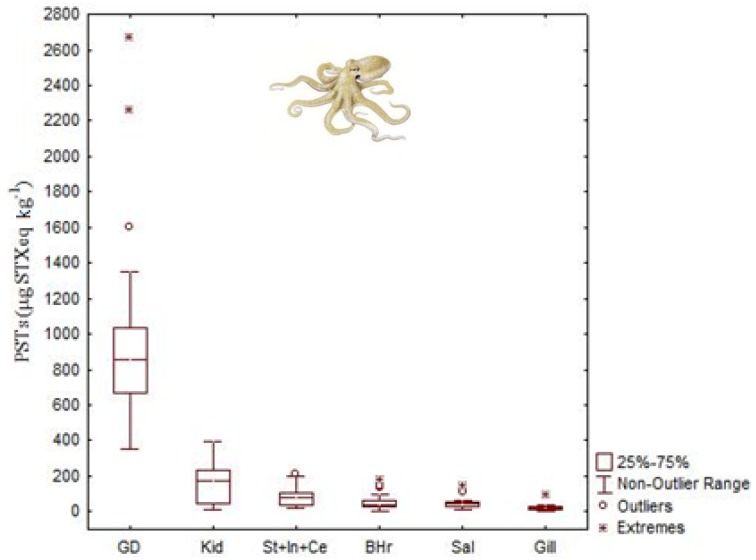
Total concentration (median, 25 and 75 quartiles, non-outlier range and outliers) of paralytic shellfish toxins (PSTs, μg STX equivalents kg^−1^) in the tissues of common octopus, *Octopus vulgaris* (data from Monteiro and Costa [45]) collected in the NW Portuguese coast. See abbreviation’s definition in the caption of Figure 7.

Studies on the accumulation and tissue distribution of PSTs in cephalopods are limited and only focused in three species, namely the common octopus (*Octopus vulgaris*), the Humboldt squid (*Dosidicus gigas*) and the Australian octopus (*Octopus* (*Abdopus*) sp. 5) [44,45,46,57] (Table 1). As with DA, PSTs accumulated to the greatest extent in DG >> kidneys > stomach > branchial hearts > posterior salivary glands > gills of *O. vulgaris* (Figure 11). Toxin concentrations, in terms of saxitoxin equivalents, ranged from 390 to 2680 μg STX equivalents kg^−1^ in the DG; from 44 to 390 μg STX equivalents kg^−1^ in the kidneys; from 21 to 210 μg STX equivalents kg^−1^ in the stomach, from 14 to 140 μg STX equivalents kg^−1^ in salivary glands and from not detected to 180 μg STX equivalents kg^−1^ in branchial hearts [45].

**Figure 12:**
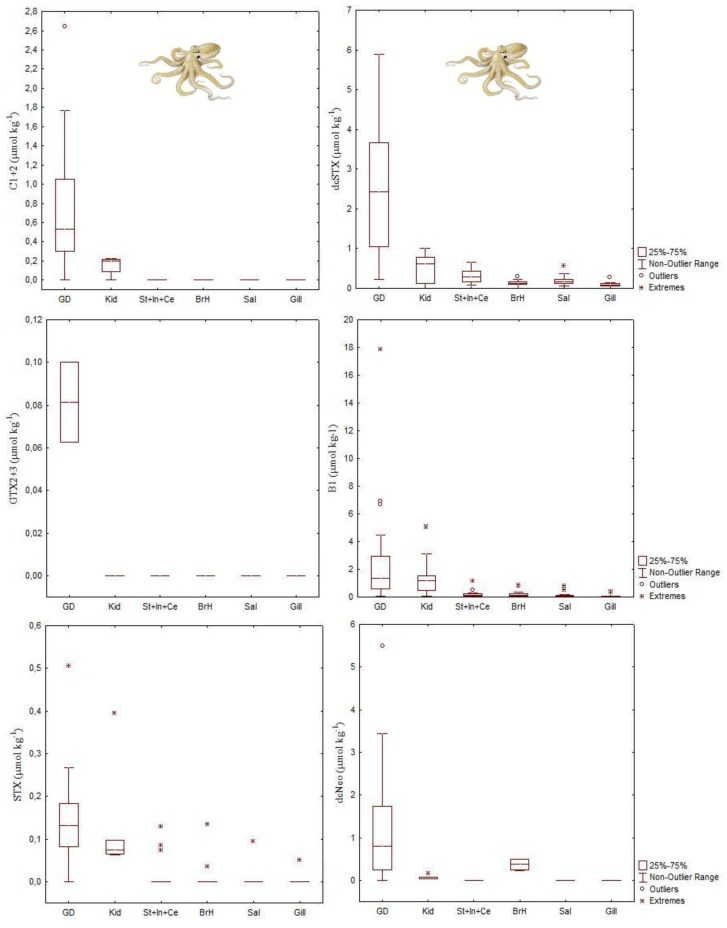
Concentration of paralytic shellfish toxins (C1 + 2 *N*-sulfocarbamoyl-gonyautoxin-2 and -3; dcSTX decarbamoylsaxitoxin; GTX2 + 3 gonyautoxin-2 and -3, B1 gonyautoxin-5 or GTX5; STX saxitoxin; NEO neosaxitoxin) in several tissues of common octopus, *Octopus vulgaris* (data from Monteiro and Costa [45]) collected from the NW Portuguese coast. See abbreviation’s definition in the caption of Figure 7.

Toxin profile of the DG of the *O. vulgaris*, collected from the Portuguese coast where *Gymnodinium catenatum* is the main PST producer, was constituted by C1 + 2, dcSTX, GTX2 + 3, B1, STX and dcNEO (Figure 12). The toxin profile of the remaining organs, with the exception of organs with excretory functions was limited to B1, dcSTX and STX. In addition to the DG, dcNEO was found in kidneys and branchial hearts. Small amounts of C1 + 2 were also present in kidneys. B1 and dcSTX were the most abundant toxins in all tissues analyzed. They comprised about 70% of the PSTs molar fraction in the DG. In the remaining organs B1 and dcSTX justified more than 90%. Decarbamoyl saxitoxin was the most abundant toxin detected in all tissues with the exception of the organs with excretory function, where B1 was the dominant toxin. Selective elimination of PSTs with higher elimination of B1 and retention of dcSTX is suggested in this study for the common octopus [45]. It is worth noting that the PSTs regulatory limit for closures to shellfish harvesting is 800 μg STX equivalents kg^−1^.

**Figure 13:**
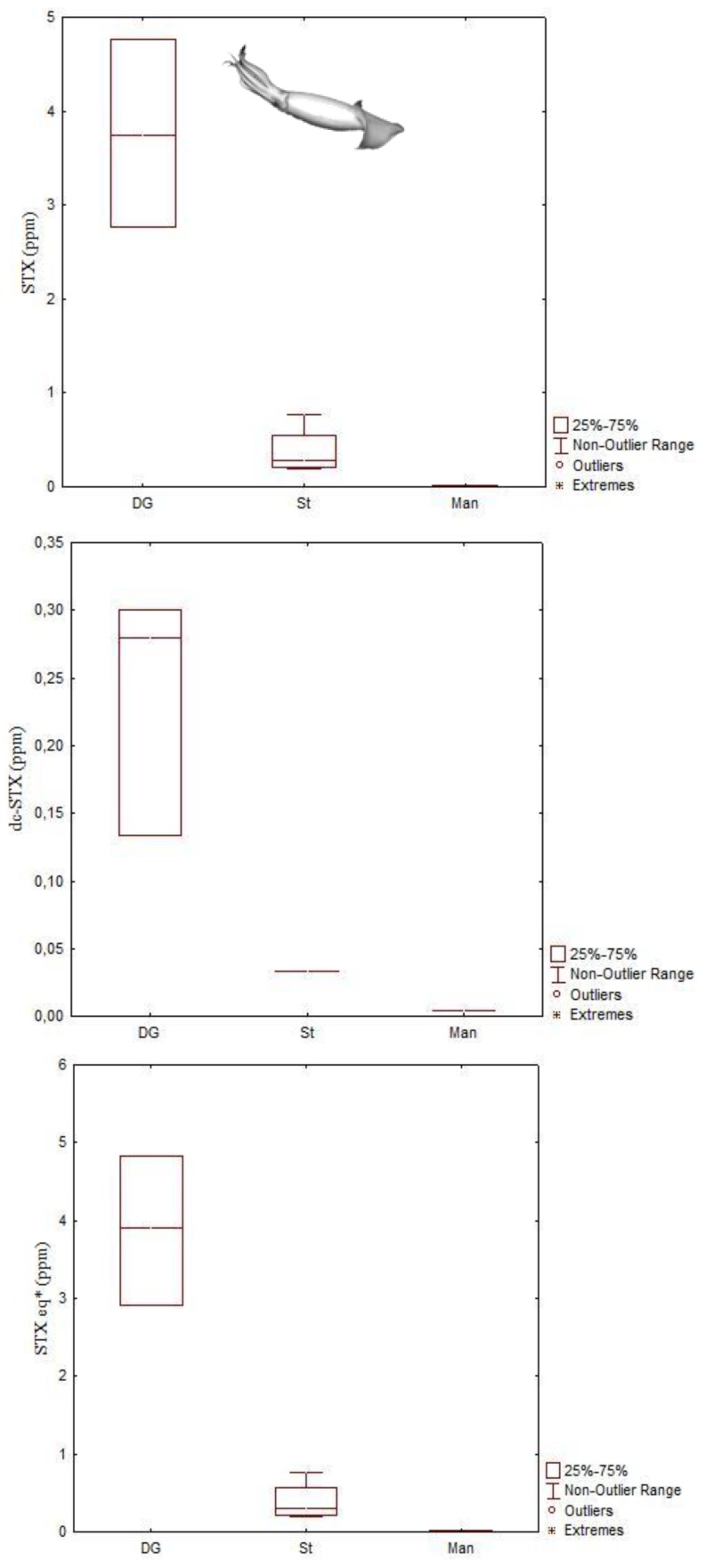
Concentrations of paralytic shellfish toxins detected in Humboldt squids (*Dosidicus gigas*) from stranding events in British Columbia, Canada. *STX equivalents = STX + dcSTX×0.51 (data from Braid *et al.* [44]).

Analyses undertaken in Humboldt squids also revealed a wide range of PST levels in both stomach and DG (Figure 13). The PST levels in the stomach ranged from 470 to 7700 μg STX equivalents kg^−1^ and in the DG ranged from 2910 to 4830 μg STX equivalents kg^−1^, the latter being up to 6 times greater than the regulatory limit of PST in shellfish. The toxin profile was predominantly STX, but low levels of dcSTX were also detected (Figure 13) [44].

Unlike *O. vulgaris* and *D. gigas*, which accumulate PSTs in their viscera (especially the DG) and not in the edible part (*i.e.*, mantle and arms), the Australian *Octopus* (*Abdopus*) sp. 5 is known to accumulate and retain STX in the arms, with no measurable levels of other derivatives. Since this study was only focused on the edible portion of the octopus, no other organs and tissues were analyzed. Here, and unlike most cases where this toxin has a dinoflagellate origin, the contamination seemed to be caused by the red algae *Jania* sp. [46]. The risk of human poisoning by the consumption of cephalopods is considerably lower for the common octopus and the Humboldt squid, since the PST storage is made exclusively in the viscera, which is usually inedible and removed for human consumption [44,45,57]. On the other hand, the consumption of the Australian octopus presents high risk of paralytic shellfish poisoning, since the allocation of the toxin is within the most edible portion of this small octopus, the arms [46].

### 4.3. Palytoxins

Currently there are not sufficient studies to determine the effect that PlTX has in marine organisms. Palytoxin, much like all other toxins previously mentioned, can be found in filter-feeding organisms and can also accumulate in their digestive organs [80,128,129,130]. During a large bloom of *Ostreopsis ovata* in Italy, many marine organisms, especially invertebrates, were greatly impacted [47]. Octopods (species not identified) were found stranded on beaches, sea urchins lost all spines, and sea stars had their arms folded. Chemical analyses confirmed that all samples were contaminated with PlTX. Among PlTXs, Ovatoxin-a (OVTX-a) was the dominant compound found. Stranded octopods revealed higher contamination levels, with ovatoxin reaching 971 μg kg^−1^ and palytoxin reaching 115 μg kg^−1^. Ovatoxin-a reached maximum concentrations of 164 μg kg^−1^ in sea urchins and of 238 μg kg^−1^ in mussels. The regulatory limit for PlTXs in seafood has recently established to be 30 μg kg^−1^ shellfish meat (EFSA—European Food Safety Authority). Thus, these findings indicated, once again, the great capacity of these organisms to accumulate toxins [47]. 

## 5. Potential Effects of HAB-Toxins in Cephalopod Early Stages

Although there are no studies available, physiological and behavioral changes are expected in the cephalopod early stages when exposed to HAB-related marine toxins, namely by: (i) Direct ingestion (in the case of exotrophic paralarvae); (ii) By diffusion through the skin or (iii) Due to parental effects (*i.e.*, spawner’s reproductive dynamics during HAB episodes). Nonetheless, studies performed in fish and crustacean larvae could give some insight on what is expected to happen in these mollusks. Early stages are more predisposed to marine phycotoxins, since they have higher mass-specific metabolic rates [131,132] and are still deprived of effective detoxification systems [74], which can transform the toxins into less toxic compounds (or even excrete them altogether). Yet, DA seems to not induce significant changes on the survival and growth of crustacean and bivalve early stages. The absence of negative effects may be due to the fact that this toxin affects the central nervous systems, and these invertebrates lack complex brains [74,133]. Moreover, when the effect of DA was tested in embryos of the freshwater zebra fish, *Danio rerio*, they showed highly reduced hatching rates and the hatchlings showed absence of touch reflexes [74,134].

Regarding OA and DTX studies, it has been shown that, in spite of the protective cyst shell, metanauplii and especially nauplii of *Artemia* presented loss of balance and thus became incapable of swimming and sunk to the bottom, which led to high mortality rates [135]. Exposure of *Artemia salina* to cultures of different species of *Alexandrium*, a STX producer, showed that the metanauplii were the most sensitive phase of the life cycle, with survival rates decreasing significantly more than other stages tested (nauplii and adults). Different crab zoea larvae showed very low survival rates when fed with *A. andersoni*. Also, the larvae presented sublethal effects, such as reduced metabolic rates. The exposure to STX also led to changes in behavior as well, with the larvae choosing not to move upward in order to avoid the positively buoyant microalgae [136,137,138]. In fish, namely in capelin (*Mallotus villosus*), Atlantic herring (*Clupea harengus*) [139] and Atlantic mackerel (*Scomber scombrus*) [59], the effect of this toxin was considerably more severe, with high mortality rates (between to 92% and 100%), paralysis and erratic movements that resulted in sinking to the bottom and led to death. Experiments conducted with zebra fish revealed that exposure of the embryos to STX caused morphological deformities, with edema in eyes, heart cavity and yolk sac. Furthermore, the swim bladder did not inflate and after four days of exposure all the larvae were paralyzed. It was also shown that the freshwater zebra fish embryos were more sensitive than the marine herring embryos [5,6,59,139]. Similar findings are expected to be observed in cephalopods as they possess a complex nervous system and highly developed brain, comparable to that of vertebrates. 

## 6. Conclusions

Cephalopods (e.g., coastal octopus and cuttlefish) are good candidates to track the occurrence of harmful algal blooms and marine toxins. Due to the fact that their complex nervous system is comparable in certain aspects to those of the vertebrates, they are excellent model organisms to study the effects HAB-toxins. At the present moment, there are no studies concerning the uptake, possible accumulation and subsequent transfer of other HAB-toxins, for example OA, DTX, PTX, YTX, AZAs, BTX and CTX. In the future, it is essential to know: (i) If they accumulate these toxins during (and after) HABs; (ii) The toxins depuration rates; and (iii) Their storage capabilities. Since these organisms have been found to accumulate considerably high levels of toxins without apparent harm, it is of great value to identify the mechanisms that provide them the ability to metabolize and detoxify HAB-toxins, which is certainly critical to their survival. These organisms have probably evolved to acquire additional resources that enable them to tolerate the HAB-toxins. The influence of HAB-toxins either in their dissolved or particulate (intracellular) form in the critical early life stages is another important issue to be investigated. Regarding food safety, with exception of the Australian octopus that has been found to accumulate STX in their arms (and the consumption of non-eviscerated juvenile octopus or cuttlefish specimens), cephalopods do not seem to represent human health hazards. 
